# Characterisation of Neutropenia-Associated Neutrophil Elastase Mutations in a Murine Differentiation Model *In Vitro* and *In Vivo*

**DOI:** 10.1371/journal.pone.0168055

**Published:** 2016-12-12

**Authors:** Michael Wiesmeier, Sanjivan Gautam, Susanne Kirschnek, Georg Häcker

**Affiliations:** 1 Institute for Medical Microbiology and Hygiene, Medical Center–University of Freiburg, Faculty of Medicine, University of Freiburg, Freiburg, Germany; 2 Spemann Graduate School of Biology and Medicine, University of Freiburg, Freiburg, Germany; 3 Faculty of Biology, University of Freiburg, Freiburg, Germany; University of the Pacific, UNITED STATES

## Abstract

Severe congenital neutropenia (SCN) is characterised by a differentiation block in the bone marrow and low neutrophil numbers in the peripheral blood, which correlates with increased risk of bacterial infections. Several underlying gene defects have been identified in SCN patients. Mutations in the neutrophil elastase *(ELANE)* gene are frequently found in SCN and cyclic neutropenia. Both mislocalization and misfolding of mutant neutrophil elastase protein resulting in ER stress and subsequent induction of the unfolded protein response (UPR) have been proposed to be responsible for neutrophil survival and maturation defects. However, the detailed molecular mechanisms still remain unclear, in part due to the lack of appropriate in vitro and in vivo models. Here we used a system of neutrophil differentiation from immortalised progenitor lines by conditional expression of Hoxb8, permitting the generation of mature near-primary neutrophils *in vitro* and *in vivo*. NE-deficient Hoxb8 progenitors were reconstituted with murine and human forms of typical NE mutants representative of SCN and cyclic neutropenia, and differentiation of the cells was analysed *in vitro* and *in vivo*. ER stress induction by NE mutations could be recapitulated during neutrophil differentiation in all NE mutant-reconstituted Hoxb8 cells. Despite ER stress induction, no change in survival, maturation or function of differentiating cells expressing either murine or human NE mutants was observed. Further analysis of *in vivo* differentiation of Hoxb8 cells in a murine model of adoptive transfer did not reveal any defects in survival or differentiation in the mouse. Although the Hoxb8 system has been found to be useful for dissection of defects in neutrophil development, our findings indicate that the use of murine systems for analysis of NE-mutation-associated pathogenesis is complicated by differences between humans and mice in the physiology of granulopoiesis, which may go beyond possible differences in expression and activity of neutrophil elastase itself.

## Introduction

Congenital neutropenias are usually monogenetic, either inherited or sporadically arising immunodeficiencies. The lack of mature, functional neutrophil granulocytes is accompanied by a high risk for bacterial infections in these patients. Several defects of individual genes have been identified, which are involved in the pathogenesis of neutropenia. The neutrophil elastase gene *ELANE* is the most frequently mutated gene and is affected by autosomal-dominant mutations in around 50% of neutropenia patients [1–3]. Other genes such as glucose-6-phosphatase-3 (G6PC3) [4], HCLS-associated Protein–X1 (Hax1) [5], the zinc finger protein Gfi-1[6] or adenylate kinase 2 (Ak2) [7] are found mutated at lower rates. Mutations in *ELANE* can either cause severe congenital neutropenia (SCN), or, depending on the type of mutation, also cyclic neutropenia where phases of reduced neutrophil counts alternate with normal blood counts. More than 100 single, mostly point mutations have been identified in *ELANE* [8]. Besides treatment with high doses of G-CSF, hematopoietic stem cell transplantation is the sole curative treatment option of severe congenital neutropenia.

The pathophysiological mechanisms of SCN are still not well understood. A maturation arrest probably occurs in the bone marrow in all cases, since typically no differentiation stages of the neutrophil lineage beyond the promyelocyte stage are found in the bone marrow of SCN patients [9]. Why later maturation stages are absent is largely unclear; it appears most likely that increased apoptosis is responsible for this defect. However, analysis of the underlying mechanisms and separation of abnormal maturation from apoptosis-induced defects has proved difficult.

NE, together with proteases of related function like protease 3 or cathepsin G, belongs to the family of serine proteases. Its expression is up-regulated during early neutrophil differentiation and the protein is stored in azurophilic granules in differentiated cells from the promyelocyte stage on, but NE is also found extracellularly either secreted or at the cell surface [10–13].

An early attempt to explain the pathophysiological mechanisms of NE mutations led to the hypothesis of disturbed vesicle trafficking, resulting in mislocalisation of neutrophil elastase [14]. This hypothesis is strengthened by the observation that dogs carrying mutations in the protein AP3b1, which is involved in intracellular vesicle trafficking and directs trans-golgi export of transmembrane cargo proteins to lysosomes, exhibit cyclic haematopoiesis [14]. In human patients, disruption of the C-terminal sorting signal of NE or failure to remove the C-terminal pro-sequence, preventing its interaction with AP3, is characteristic for most *ELANE* mutations found in SCN patients. This results in misdirection to the membranes, whereas mutations predominantly found in cyclic neutropenia favour trafficking to granules (Horwitz 2013). Further reports have additionally implicated ER stress and unfolded protein response (UPR) in the emergence of SCN [15, 16]: accumulation of unfolded or misfolded NE proteins in the ER is believed to induce ER stress, resulting in induction of the unfolded protein response (UPR). Previous analyses indicate that NE mutations can lead to misfolding of the protein and subsequent induction of UPR which then results in enhanced apoptosis [15, 17, 18]. However, certain mutations (found both in cyclic neutropenia and SCN patients) affecting the translational start site, generate N-terminally truncated variants without ER translocation signal sequence. Since these mutated proteins are very likely to induce misfolding and ER stress, the UPR hypothesis appears unable to explain the phenotype in all cases [18].Although both the mislocalisation and the ER stress hypotheses are plausible and complement each other in certain respects, they are not able to comprehensively and satisfactorily explain pathogenesis of neutropenia.

Two knock-in mouse models reflecting two different mutations causing SCN in humans have been established to date [17, 19]. In contrast to the human situation, neither NE-V72M knock-in [19] nor NE-G193X knock-in mice [17] led to neutropenia. Although neutropenia could be provoked in G193X-knock-in mice under conditions of *in vivo* proteasome inhibition, basal and emergency granulopoeisis were not disturbed [17]. Human *in vitro* differentiation models based on conventional human cell lines also cannot satisfactorily rebuild the situation of neutrophil differentiation and have been of limited value so far. Only the recent establishment of human *in vitro* myelopoiesis models using patient-derived iPS cells seems to represent a promising novel approach for analysis of the mechanisms of human SCN disease in vitro and for attempts to correct the disease [20–22].

Using this approach, Hiramoto et al. [20] showed reduced expression of members of theWnt3a/β-catenin pathway in NE mutant cells and restoration of neutrophil maturation by Wnt3a treatment *in vitro*. Another iPS cell-based study showed that low-dose G-CSF in combination with a small molecule NE inhibitor rescued neutrophil maturation, restored CEBPα, but not CEBPβ expression, corrected mislocalisation of mutated NE and ameliorated induction of ER stress [22]. Although very promising for future *in vitro* analysis, iPS cell-based human systems still have certain drawbacks such as the low cell numbers available and restriction to *in vitro* analysis.

We here investigated the suitability of a model of mouse neutrophil differentiation for the analysis of NE-deficiency. The model is based on conditional expression of Hoxb8 fused to the ER receptor [23] and allows for establishment of progenitor cells derived from murine bone marrow by retroviral transfer and subsequent selection of cells in the presence of specific cytokines. Progenitors can be differentiated within few days into morphologically and functionally mature neutrophils *in vitro*, which are almost indistinguishable from primary mouse neutrophils. This model enables production of large cell numbers and allows for genetic modification by retro- and/or lentiviral gene transfer [23, 24]. Moreover, analysis of *in vivo* differentiation is possible by adoptive transfer of Hoxb8 progenitors into mice [25, 26].

We here use the Hoxb8 model to reconstitute progenitors of the neutrophil lineage with human and murine NE mutants typical for SCN or cyclic neutropenia, respectively. Granulopoiesis in these cells is characterised with regard to survival, differentiation course and functionality both *in vitro* and *in vivo*.

## Results

### ER-stress induction by expression of human and mouse NE mutants in HEK 293FT cells

Two different representative mutations found in human congenital neutropenia patients were chosen for analysis. Both are single base amino acid missense mutations typical for SCN and cyclic neutropenia, respectively. The mutation G185R [27–29] is found in SCN patients with a particularly severe SCN phenotype of neutropenia with an absolute neutrophil count close to zero, poor response to G-CSF treatment and frequent occurrence of myelodysplastic syndrome or acute myeloid leukemia [2]. The mutation S97L in contrast is found mainly in cyclic neutropenia patients [27, 28] but can also cause SCN in some cases [30]. The two characteristic mutations G185R and S97L were introduced in both human and murine NE cDNAs.

**Fig 1:**
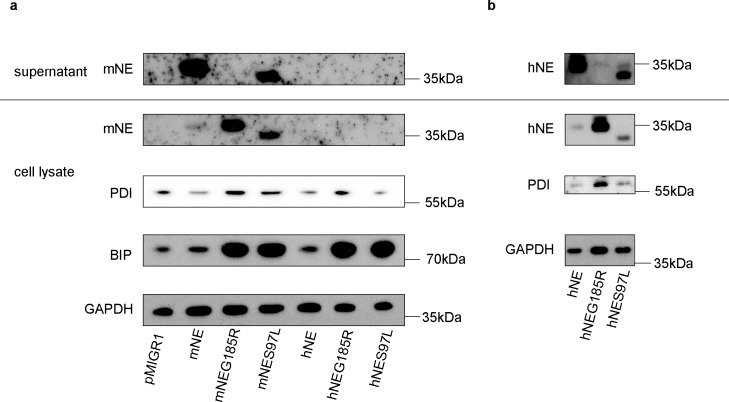
NE expression, secretion and ER stress induction in transfected 293FT cells. Supernatants and cell lysates from 293FT cells were analysed 64h after transfection with either empty vector (pMiGR1), murine neutrophil elastase (mNE), human neutrophil elastase (hNE) or mNE/hNE mutants G185R (mNEG185R/hNEG185R) or S97L (mNES97L/ hNES97L). (A) expression of murine neutrophil elastase (mNE) and the ER-stress markers BIP (GRP78/HASP5) and PDI in cell lysates and supernatants, (B) expression of human neutrophil elastase (hNE) in cell lysates and supernatant (the same samples as in (A) were rerun on a separate gel and probed with a human-specific anti-hNE antibody). Samples corresponding to 20μg cell lysate or 20μl of supernatant were separated by SDS-PAGE, transferred onto nitrocellulose membranes and probed for the antibodies indicated. GAPDH served as loading control. Data represent one of two independent experiments.

NE wt and mutants were first transfected into HEK 293FT cells. Transiently expressed human and mouse NE could be readily detected by Western blot in whole cell lysates of HEK 293FT cells (Fig 1A and 1B; S7 and S8 Figs). We tested three different commercially available antibodies with varying results, see S6 Fig. While the NE G185R showed the same apparent size as wt NE in SDS-PAGE, the NE S97L mutant migrated at a lower molecular weight, which is probably due to altered glycosylation, as described before [15]. For all tested constructs, transfected 293FT cells remained viable with a cell death rate below 10% and with a transfection efficiency of around 60–70% as determined by GFP-positivity (see S5 Fig).

Wt NE was secreted efficiently into the supernatant while the mutants were retained either partially (S97L) or completely (G185R) in the cells; this pattern was the same for hNE and mNE. This is in agreement with published data [15]. Correlating with accumulation of NE protein in the cells, we observed an upregulation of the ER stress marker BIP in cells expressing the G185R or S97L mutants (Fig 1A). Blots were reprobed for PDI, an additional ER stress marker. PDI was clearly induced in cells expressing the human or murine G185R mutant in comparison to the wt NE. Upon expression of the murine or human mutants S97L, induction of PDI was seen in most experiments, but results were less consistent than with the G185R mutant.

### Proliferation, survival, ER stress and maturation of mNE mutant-reconstituted Hoxb8 neutrophils during differentiation

Hoxb8 neutrophil progenitor cells from NE-/- murine bone marrow cells (129/Sv background) [31, 32] were established by retroviral transduction of conditional estrogen-regulated Hoxb8 followed by selection of a polyclonal progenitor cell line in the presence of stem cell factor (SCF) [23]. NE-deficient Hoxb8 neutrophil progenitors were then reconstituted by retroviral transduction with wt or mutant murine NE constructs carrying an IRES-EGFP as a marker. The transduced polyclonal cell population was cell-sorted for EGFP-positivity.

**Fig 2:**
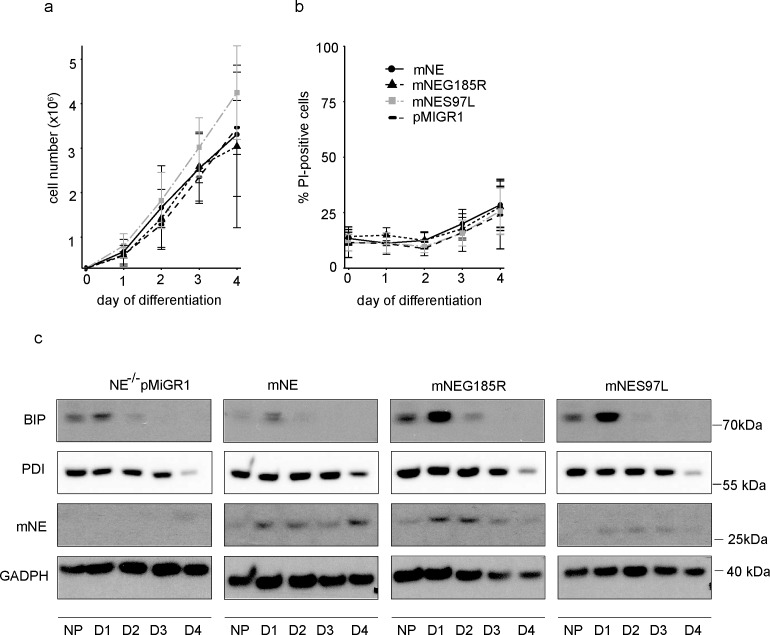
Characterisation of differentiating Hoxb8 cells reconstituted with murine wt or mutant NE. NE^-/-^ Hoxb8 neutrophils transduced with empty vector pMIGR1 (pMiGR1), murine neutrophil elastase (mNE), or mNE mutants G185R (mNeG185R) or S97L (mNeS97L) (genetic background 129/SV) were induced to undergo differentiation by estrogen withdrawal for 4 days. (A) Proliferation of differentiating neutrophils as measured daily by manual counting (Neubauer chamber) after initial seeding of 300,000 cells/well in a 6-well plate at day 0. Data are mean/SD of 3 independent experiments. (B) Cell death analysis of Hoxb8 neutrophils cultured from day 0 to day 4 of differentiation as above. Cell death was determined by propidium iodide (PI) staining for loss of cell membrane integrity. Shown are % PI-positive cells. Data are mean/SD of 4 independent experiments. (C) Western blot showing murine neutrophil elastase (mNE), BIP (GRP78/HASP5) and PDI in cell lysates of murine Hoxb8 progenitors (NP) and day 1 to day 4 (D1-D4) in vitro differentiated neutrophils. Samples were separated by SDS-PAGE, transferred onto nitrocellulose membranes and probed with antibodies against mNE and BIP followed by detection using enhanced chemoluminescence. GAPDH served as as loading control. Data shown are representative of 2 independent experiments.

Progenitor cultures were without any apparent abnormalities and showed normal proliferation and survival of progenitors (data not shown). Neutrophil differentiation was induced by estrogen withdrawal. Absolute cell numbers increased during differentiation from 300,000 to an average of 3–4 Mio per well. No striking differences between cell lines were seen in terms of absolute numbers during differentiation and in differentiated cells, with slightly higher cell numbers of S97L mutants (Fig 2A), and cultures showed comparable survival during differentiation of the lines expressing the various constructs (Fig 2B).

Transduced murine NE could be detected by Western blot. Higher expression of NE protein was seen early during differentiation compared to the progenitor stage; this was similar for wt and mutant NE forms (Fig 2C), and protein levels appeared to be regulated similarly to endogenous murine NE in WT Hoxb8 cells (see S11 Fig). This points to an involvement of posttranslational mechanisms in the control of NE protein levels during neutrophil differentiation. The ER-stress marker BIP was detected in NE-deficient cells at progenitor stage and on day 1 of differentiation, where BIP expression was transiently, probably physiologically, induced. This was essentially unaffected by the expression of wt mNE. The expression of either mutant caused the appearance of a substantially stronger BIP-signal, which however also appeared only transiently (Fig 2C). Slightly increased PDI expression compared to wt reconstituted cells was seen in progenitors and early in differentiation in G185R mutant cells but was not visible in S97L expressing cells, indicating milder ER stress induction in case of the S97L mutant. The ER stress induced by expression of NE mutants was thus recapitulated in differentiating murine neutrophils but did not seem to have an impact on proliferation or survival during neutrophil differentiation in vitro.

The differentiation course of murine progenitors reconstituted with NE mutants was further assessed by analysis of surface marker expression and morphology.

**Fig 3:**
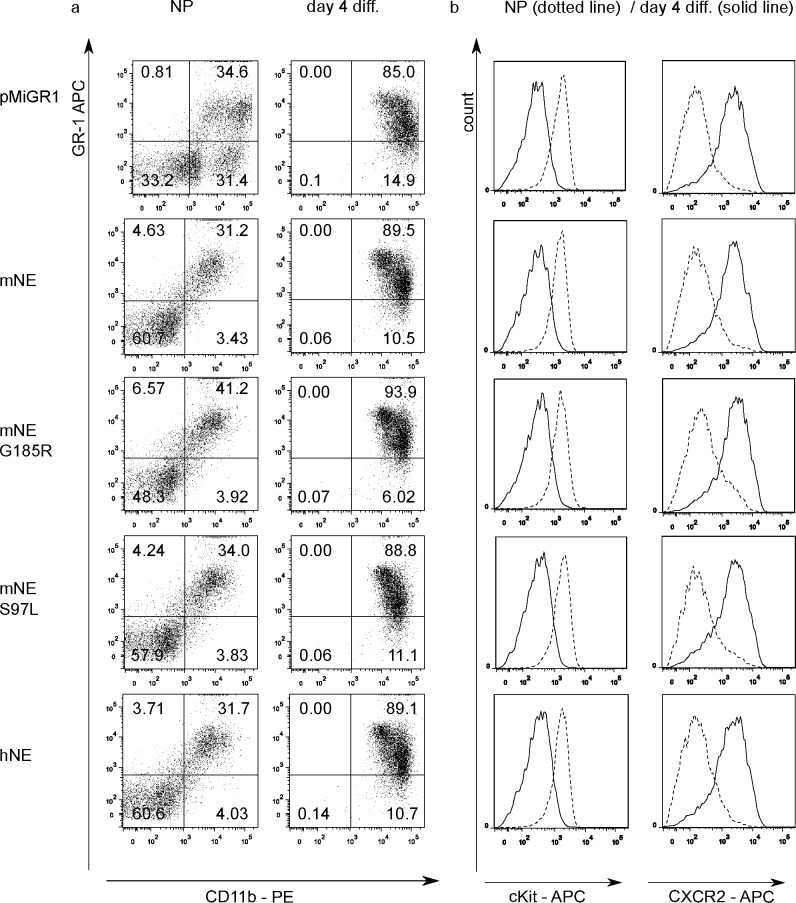
Surface expression of myeloid differentiation markers on progenitors and differentiated neutrophils. Murine Ne^-/-^ progenitors (NP) and day 4 differentiated neutrophils (genetic background 129/Sv) transduced with empty vector (pMiGR1), mNE, hNE, mNE mutants G185R (mNeG185R) or S97L (mNeS97L) were stained with fluorescence-conjugated antibodies against Gr-1, CD11b, c-kit and CXCR2 and analysed by flow cytometry. (A) Gr-1-APC/CD11b-PE double stained NP/day 4 diff. neutrophils. Data are representative of two (NP) or 4 (day 4 diff.) independent experiments. (B) Histograms of NP (dotted line) and day 4 differentiated neutrophils (solid line) showing expression of c-kit and CXCR2. Data are representative of two independent experiments.

The upregulation of CD11b and Gr-1, downregulation of c-kit and upregulation of CXCR2, which is characteristic for neutrophil development and is also observed during differentiation of Hoxb8 neutrophils[23, 26], was unchanged in mNE mutant compared to wt mNE or control NE-deficient cells (Fig 3A and 3B). Giemsa-stained progenitors and day 4 differentiated cells displayed normal differentiation by morphology (S1 Fig). The data therefore give no indication of a differentiation block or lack of differentiation.

### Cytokine secretion in murine NE mutant-reconstituted Hoxb8 neutrophils

**Fig 4:**
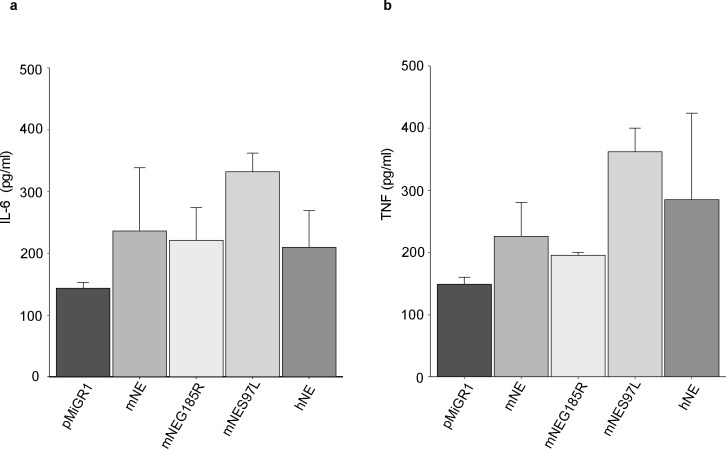
Cytokine secretion by day 4 differentiated Hoxb8 neutrophils upon LPS stimulation. NE^-/-^ Hoxb8 cell lines transduced with empty vector pMIGR1 (pMiGR1), murine neutrophil elastase (mNE), human neutrophil elastase (hNE), or mNE mutants G185R (mNeG185R) and S97L (mNES97L) (genetic background 129/Sv) were differentiated for 4 days and then stimulated with 1 μg/ml LPS. (A, B) The cytokines IL-6 (A) and TNF (B) were measured by ELISA in supernatants harvested after 16h of LPS-stimulation. Data are mean/SD of three independent experiments and dual ELISA of each stimulation. Unstimulated medium control values are 0-6pg/ml.

To test for function of NE mutant-reconstituted neutrophils, cytokine secretion in response to proinflammatory stimulation was analysed. TNF and IL-6 were measured by ELISA in supernatants of day 4 differentiated, wt mNE or mutant mNE-reconstituted neutrophils stimulated with LPS. No striking differences were seen; mutant and wt NE as well as empty vector control cells readily responded to this stimulus. Murine NES97L-reconstituted cells showed slightly, but not significantly increased IL-6 and TNF secretion (Fig 4).

ER-stress is a known consequence of the NE-mutations, and one hypothesis of neutropenia-development is ER-stress induced apoptosis [15, 16]. Since no spontaneous apoptosis was detected despite ER-stress induction we tested whether cells expressing mutant NE were more sensitive to apoptosis-induction by the ER-stressor tunicamycin. Cells were treated with tunicamyin on day 1 of differentiation, when signs of ER stress induction were strongest, or in differentiated neutrophils (day 4). No enhanced susceptibility to cell death was seen in cells expressing the mNEG185R or mNES97L (S2 Fig and data not shown), and no induction of cell death was observed when cells where LPS stimulated (data not shown), which has been described to induce ER stress in the context of inflammatory responses [33].

The data obtained so far argue for normal differentiation, survival and function of mouse neutrophils and progenitors reconstituted with murine NE mutants *in vitro*. This to a large degree confirms data from previous studies, in particular where the knock-in of human SCN-mutations in the mouse *Elane* gene failed to recapture SCN. There were however two aspects that we had not considered so far. First, there is the possibility that mutations in the human *ELANE*gene may cause a different phenotype from the experimental ones in mouse *Elane*. Secondly, there was the possibility that differentiation *in vivo*, which takes place in the complex situation of the bone marrow compartment, may require additional signals than can be provided in the cell system *in vitro*.

To test for these two aspects we generated mouse progenitor cells (based on the gene-knock-out) transgenic for hNE. The *in vivo*-model is on the C57BL/6 background, and we therefore established neutrophil progenitors based on NE-/- cells (C57BL/6 background), reconstituted with the NEG185R mutation found in SCN patients with severe disease.

### *In vitro* analysis of hNE-reconstituted Hoxb8 cells on C57BL/6 background

**Fig 5:**
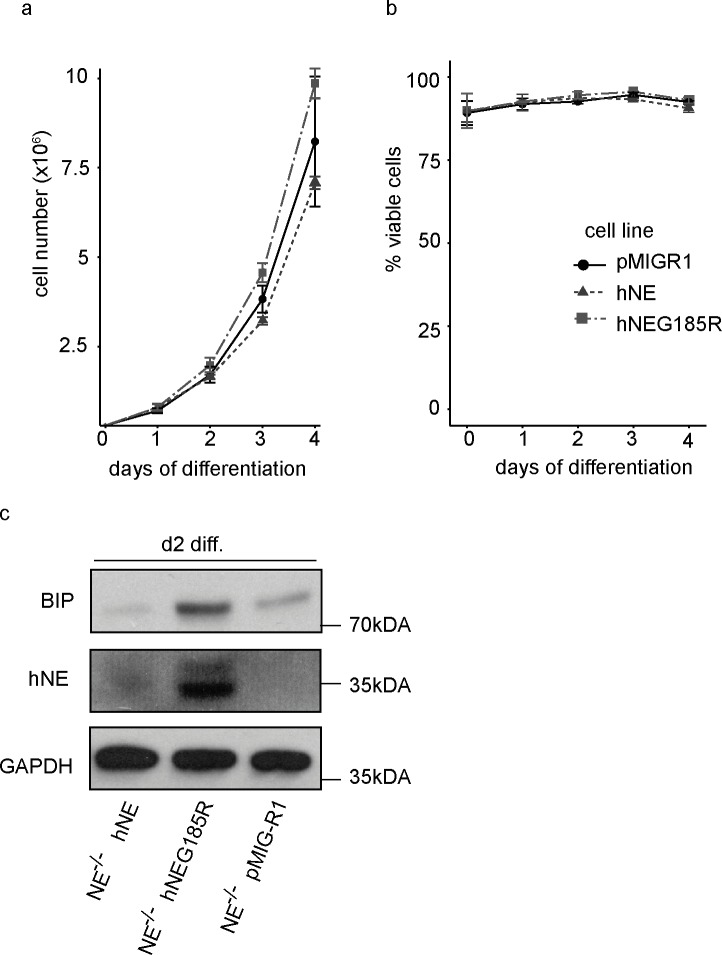
Characterisation of differentiating Hoxb8 cells reconstituted with human wt or mutant NE. NE^-/-^ Hoxb8 neutrophils transduced with empty vector pMIGR1 (pMiGR1), human neutrophil elastase (hNE), or hNE mutant G185R (hNEG185R) (genetic background C57BL/6) were induced to undergo differentiation by estrogen withdrawal for 4 days. (A) Cell numbers of differentiating neutrophils were measured daily by automated counting with a CASY cell counter after initial seeding of 300,000 cells/well in a 6-well plate at day 0. Data are mean/SD of 3 independent experiments. (B) Cell death analysis of Hoxb8 neutrophils cultured from day 0 to day 4 of differentiation as described above. Viability was determined using a CASY cell counter. Data are mean/SD of 3 independent experiments. (C) Western blot analysis of human NE (hNE) and BIP (GRP78/HASP5) expression in cell lysates of Hoxb8 cells at day 2 of differentiation. Hoxb8 cells transduced with empty vector (pMIGR1), human wt NE (hNE) or human NE mutant G185R (hNEG185R) were induced to undergo differentiation *in vitro* by estrogen withdrawal. Differentiating cells were harvested on day 2. Samples were directly lysed in Laemmli buffer and heated at 95°C for 5 min. Samples were separated by SDS-PAGE, transferred onto nitrocellulose membranes and probed with antibodies against mNE and BIP followed by detection using enhanced chemoluminescence. GAPDH served as loading control.

NE^-/-^, hNEG185R-reconstituted Hoxb8 cells cultivated as neutrophil progenitors behaved normally in terms of proliferation, survival and morphology (S3 Fig and data not shown). When neutrophil differentiation was started by estrogen withdrawal, cells had normal proliferation kinetics (Fig 5A) and did not show any compromised survival during differentiation (Fig 5B). Analysis of protein expression showed retention of the hNE mutant G185R in the cell (Fig 5C), and detection of the ER stress marker BIP revealed again induction of ER stress by the mutant G185R, but not by wt hNE (Fig 5C). These data parallel the findings with the mutant mouse protein shown above.

Characterization of neutrophil differentiation of the hNEG185R mutant compared to wt hNE and empty vector control by means of morphology and surface marker analysis revealed no abnormalities (S3 Fig), indicating that, as had been seen with the murine form of the protein, expression of human NE mutants in mouse cells does not impair neutrophil development or survival.

So far all reconstitutions were done on a NE-deficient background. Since most reported NE mutations in SCN patients are mono-allelic, the question arises whether presence of the wt protein might be required for development of neutropenia. To test for such a possible requirement, we expressed the human or the murine NE G185R mutant, on the B6 wt background under conditions of endogenous expression of murine NE. The established progenitor cell lines behaved normally in terms of survival and proliferation. Survival of differentiating cells was not impaired (less than 10% cell death at day 2 and less than 20% cell death at day 4 of differentiation for wt as well as mutant-expressing cell lines), and no differentiation defects were seen as determined by surface marker analysis and cellular morphology (S4 Fig and not shown). Our data thus do not point to an involvement of the *ELANE* wt allele in SCN pathogenesis.

### *In vivo* analysis of hNE-reconstituted Hoxb8 cells

We then tested the differentiation capacity of the mutant cells *in vivo* by adoptive transfer of progenitors into lethally irradiated mice. When Hoxb8-progenitor cells are transferred into mice, progenitors differentiate and mature neutrophils can be detected in bone marrow, peripheral blood and organs [25, 26]. Following irradiation with a myelo-ablative dose, mice were reconstituted with Hoxb8 progenitors together with syngeneic bone marrow cells at a ratio of 10:1. In this model, low numbers of mature Hoxb8 neutrophils start to appear at day 4 post transplantation, and highest numbers are seen in blood and bone marrow around day 6 after transplantation [25, 26].

Lethally irradiated mice were transplanted with Hoxb8 progenitors reconstituted with either the SCN mutant hNEG185R, with wt hNE or with empty vector control. All cell lines co-expressed EGFP from an IRES as a marker permitting to trace the cells in the mouse. Mice were sacrificed at day 6 followed by analysis of blood, bone marrow and spleen.

**Fig 6:**
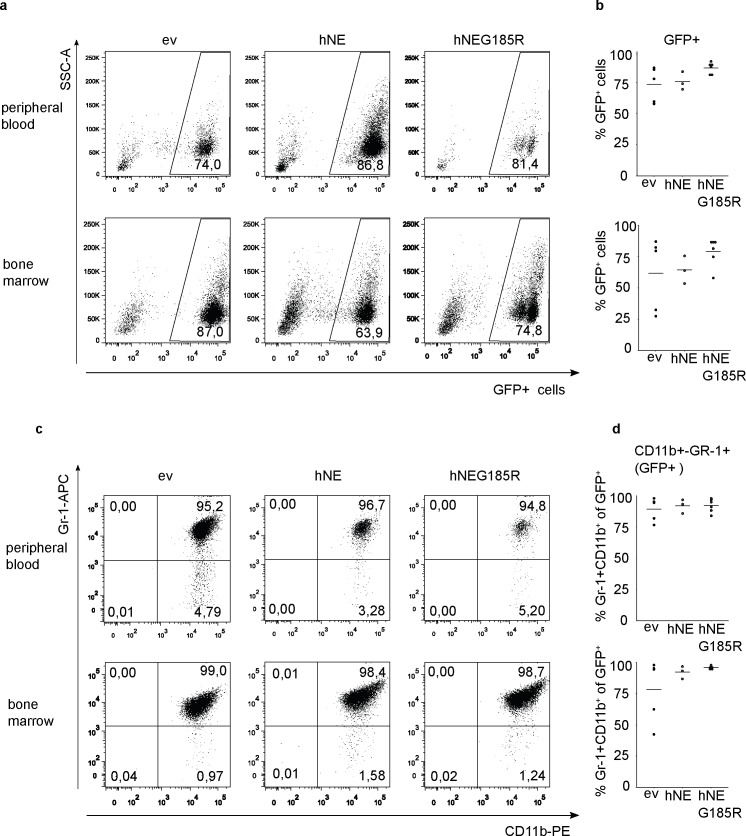
*In vivo* differentiation of Hoxb8 cells. (A-D) Flow cytometry analysis of samples from bone marrow and peripheral blood on day 6 after transplantation of irradiated mice with Hoxb8 Ne^-/-^ progenitors (C57BL/6 background) transduced with empty vector pMIGR1 (ev), human neutrophil elastase (hNE) or human neutrophil elastase mutation G185R (hNEG185R). (A) Representative FACS plots of peripheral blood and bone marrow samples analysed for GFP positivity after gating on live, DAPI-negative cells. (B) Quantitative analysis of all samples from peripheral blood and bone marrow analysed as in (A). (C) Representative FACS plots of peripheral blood and bone marrow samples showing percentage of Gr-1^+^CD11b^+^ cells of all live GFP-positive cells. (D) Quantitative analysis of Gr-1 and CD11b expression of all samples from peripheral blood and bone marrow analysed as in C. Percentage of Gr-1^+^CD11b^+^ cells of total live GFP-positive cells is shown. Each symbol represents one mouse. Horizontal bars: mean of each distribution. Data were obtained from two independent *in vivo* experiments including a total of n = 5 mice for empty vector control, n = 3 for hNE and n = 6 for hNEG185R.

GFP^+^ transplanted Hoxb8 cells were found in all organs analysed, both in mice reconstituted with the NEG185R mutant cells and in mice reconstituted with wt NE or empty vector control expressing cells. In the blood, the GFP^+^ cells constituted the majority of cells, with no significant differences between the cell lines transferred. Most of the GFP^+^ cells in the blood were Gr-1^+^CD11b^+^ mature neutrophils (ca. 95%, Fig 6).

In the bone marrow, around 60–80% GFP^+^ cells were found. Although there was some variation between animals, no obvious difference in the populations of wt (NE-deficient) cells or cells expressing wt or mutant NE was observed. The large majority of GFP^+^ cells represented mature neutrophils also in the bone marrow as judged by CD11b/Gr-1 positivity (Fig 6A–6D). Hence, the capacity of progenitors expressing human mutant NE to differentiate into mature neutrophils *in vitro* and *in vivo* was comparable to that of NE-deficient control cells as well as to wt NE-reconstituted cells. The observed induction of ER stress thus did not affect differentiation of mouse cells expressing the human version of the SCN mutant NEG185R, at least by the parameters tested.

## Discussion

Here we investigated the impact of NE mutations that are typical of human congenital neutropenia in a murine model of in vitro and in vivo neutrophil differentiation. To date, SCN caused by mutations in *ELANE* or in the Hax1 gene could not be recapitulated in mice [19] [17, 34], whereas deficiency of the G6PC3 gene in the mouse reflected the human disease well [26] [35]. In our murine Hoxb8 model, induction of ER stress and the UPR, which has been suggested as the cause for the defects resulting in neutropenia in human patients, could be recapitulated during differentiation in cells reconstituted with mutant NE. However, neither a defect in proliferation or survival of cells nor in maturation of differentiating neutrophils could be observed either *in vitro* or *in vivo*. This is in part in line with the study by Massullo et al., who expressed the same mutation we used (hNEG185R) and reported that despite accelerated apoptosis in differentiating human HL60 cells, differentiation itself was not disturbed [29]. However, the murine *in vivo* differentiation system is limited to short term differentiation analysis since transferred progenitors differentiate within a few days upon estrogen withdrawal in the context of adoptive transfer. The approach therefore may not reveal potential more subtle effects that may only appear over longer time periods or upon serial repopulation. Still, all previous and current attempts to use mouse models for NE mutation-induced neutropenia have failed to reproduce the phenotype observed in humans.A possible explanation for the lack of impairment of survival and of differentiation block in our Hoxb8 model, despite the clear reproduction of ER stress, could be the existence of fundamentally different hematopoiesis regulation in humans versus mice.

Different regulation of expression and/or activity of the human compared to the murine NE might play a role and could in part explain the differences between humans and mice. A direct comparison of endogenous protein expression levels between human and murine cells is hardly possible. In our model we saw no differences between human and murine versions of NE having the same mutations in terms of survival, proliferation or maturation. However, transgenic expression of human and mouse versions occurred from the same retroviral promoter, and the system thus does not take into account possible transcriptional regulation by the endogenous promoter or potential 3’ regulatory elements in the mRNA. However, dysregulation of transcription of ELANE and of transcription factors involved herein have been considered to be part of the pathogenesis [36, 37]. In particular, our system does not take into account the possible involvement of certain transcription factors in regulation of mutated elastase, notably LEF1, which had been shown to be dysregulated in congenital neutropenia[36]. Still, differences at the protein level regarding protein stability or activity should be retained in our system in this situation.

Nanua et al. (2011) observed that levels of NE activity in murine bone marrow cells were less than 5% of that of human bone marrow, which might lead to weaker UPR activation. We did not observe differences in the capacity to induce ER stress between our murine and human variants, but since we did not compare directly the activity of mouse and human NE expressed in our system, we cannot completely exclude such differences in activity. Reconstitution of mouse cells with the human NE version under its endogenous promoter might be valuable to better mimic the human system in the mouse.

One substantial difference between the mouse models and human patients is the lack of genetic variation in the mouse. All mouse models have so far been created on either an inbred or mixed background, with low genetic variability between mice. In contrast, with the much higher genetic diversity in the human population, NE mutations could possibly be present at a certain frequency in healthy humans. Interplay of NE mutants with unrelated, yet unidentified genes might be crucial, where occurrence of additional mutations in other genes could trigger disease development. This would also explain the varying disease severity caused by certain NE mutations, ranging from relatively mild symptoms of cyclic neutropenia to SCN. Genomic information of a much larger part of the healthy population will presumably be needed to clarify this issue.Another possibility is that the effect is not strictly cell-autonomous: non-neutrophil cells expressing neutrophil elastase and affected by the mutants might additionally play a role in disease development. In order to investigate possible effects of NE expressed in non-neutrophil cells, adoptive transfer of mutant or wt hNE expressing Hoxb8 progenitors into mice on a NE mutant or KO background could be interesting.

In contrast to mutations in other genes occurring in human SCN patients, heterozygous dominant negative mutations are usually found if the NE gene is affected. It seems unclear whether normal NE expression and function is necessary for human myelopoiesis, and is disturbed in the presence of mutant NE, or whether the mutant protein leads to survival and/or differentiation defects independently of the NE protein. Nevertheless, mutant NE and induction of UPR seem to be causal for disease formation in the human neutrophil lineage, but does not seem to have an impact on neutrophil development in the mouse.

Taken together, our data as well as findings by others indicate that a likely explanation for the failure to recapitulate the human disease in the mouse system could be yet uncovered differences in the physiology of neutrophil development between the human and the mouse system, which may go beyond possible differences in expression and activity of the neutrophil elastase protein itself. However, with its flexibility quickly to test a number of mutations, the possibility to test large numbers of cells and to assess differentiation *in vitro* and *in vivo*, the Hoxb8-system is still a valuable tool for the assessment of the effect of genetic variations on neutrophil differentiation and function, and our results add our knowledge of such effects, even though no specific phenotype was seen.

A suitable human cell system, which allows more physiological in vitro differentiation than conventional cell lines and where knock-outs and knock-ins of wt and mutant NE can be performed, will probably be the best way for addressing the unsolved questions in vitro. First promising approaches in this direction have been made using iPS cells from SCN patients [20, 22]. Such iPS cell-based approaches will be a valuable tool for in vitro analysis of the pathophysiology of SCN, but given the complexity of in vivo myelopoeiesis, there will still be a need for a “humanized” mouse model of SCN.

## Material and Methods

### Cell lines and cell culture

Hoxb8 neutrophil progenitors were established from bone marrow of NE^-/-^ or wt mice on either 129/Sv (kindly provided by Dieter Jenne, München, Germany) or C57BL/6 background (kindly provided by Azzaq Belaaouaj, Reims, France). Polyclonal cell lines were derived by retroviral transduction of Hoxb8 and selection in the presence of β-estradiol and SCF, as described [23, 24]. Precursor cell lines were maintained in Optimem medium (Invitrogen, Karlsruhe, Germany) supplemented with 10% FCS (PAA, Coelbe, Germany), 30 mM β-mercaptoethanol (Sigma, Munich, Germany), antibiotics (100 IU/ml penicillin G and 100IU/ml streptomycin sulfate, PAA), 1% supernatant from SCF-producing Chinese Hamster Ovary cells (kindly provided by Hans Häcker) and 1 μM β-estradiol (Sigma-Aldrich). Differentiation was induced by removal of estrogen in the presence of SCF (without addition of G-CSF) [23]. Staining for cellular and nuclear morphology was performed on cytospins from cultures of progenitors or day 4 differentiated neutrophils by incubation with Giemsa solution (Merck, Darmstadt, Germany) after methanol fixation. Analysis was performed by brightfield microscopy at a magnification of 63x.

HEK 293FT cells obtained from Invitrogen (Invitrogen) were cultured in DMEM supplemented with 10% FCS (Pan Biotech, Aidenbach, Germany, 50 mM β-mercaptoethanol and antibiotics (100 IU/ml penicillin G and 100IU/ml streptomycin sulfate, PAA).

In some experiments, cells were treated with LPS or Tunicamycin (both from Sigma-Aldrich, Steinheim, Germany).

### Cloning of NE mutants

Human or murine NE was PCR-amplified from cDNA derived from human or murine bone marrow and was cloned into pENTR-SD/D-TOPO vector according to the manufacturer’s instructions (Invitrogen). The cloned NE coding sequence corresponded to the precursor protein. NE mutations G185R and S97L were introduced by site-directed mutagenesis using the Quick Change II site-directed mutagenesis kit (Agilent Technologies, Santa Clara, CA, USA) according to the manufacturer’s instructions. Wt and mutant NE coding sequences were then transferred into the retroviral expression vector MIGR1-GW by gateway-based LR recombination reaction (Invitrogen).

### Transient transfection of NE mutants in HEK 293FT cells

HEK 293FT cells were seeded at 500.000 cells per well in a 6 well plate. pMIGR1-GW expression constructs of murine and human wt NE and NEG185R and NES97L mutants were transfected into HEK 293FT cells using Fugene. pMIGR1 was used as empty vector control. Cells were analysed 40–64 h after transfection as indicated.

### Retroviral transduction of neutrophil precursor cell lines

To generate Hoxb8 precursor lines stably reconstituted with murine and human NE mutants or wt NE, cells were retrovirally transduced with the corresponding MIGR1-GW constructs. Retroviral particles were produced by transient transfection of the retroviral expression constructs together with the packaging plasmid pCLEco into the ecotrophic packaging cell line Phoenix using Fugene HD transfection reagent (Promega, Madison, WI, USA). Retroviral supernatants were harvested at day 2 and 3, filtered through 0,45 μm membranes and used for infection of target cells in a 12 well plate at a density of 1 x 10^5^ cells/ml in the presence of 5 μg/ml polybrene. Transduced cells were sorted for GFP-positivity by flow cytometry, and precursor lines containing >90% of GFP-positive cells were used for experiments.

### Cell death assays

Cell death was assessed by propidium iodide staining (1 μg/ml, Sigma) for loss of cell membrane integrity followed by flow cytometry analysis.

In some experiments, cell death was quantified using the CASY TT cell counter (Omni Life Science, Bremen, Germany).

### Immunoblotting

Cell culture supernatants of HEK 293FT cells were collected and centrifuged to get rid of potentially remaining cells. After adding 6x Laemmli buffer to obtain a final concentration of 1x Laemmli, the samples were immediately boiled for 5 min at 95°C. HEK 293FT cells were washed with PBS, harvested by trypsinisation, extracted in cell lysis buffer (NEB, Cell Signalling, Danvers, MA, USA) supplemented with complete protease inhibitor cocktail (Roche) on ice and boiled in Laemmli buffer at 95°C for 5 min. Hoxb8 cells were washed with PBS and directly lysed in 1x Laemmli buffer (3x 10^6^ cells/50μl) and boiled at 95°C for 5 min. Cell extracts were separated by SDS-PAGE and proteins were transferred onto PVDF membranes. Antibodies used were specific for human NE (Santa Cruz Biotechnology Inc., Santa Cruz, CA, USA, polyclonal from goat, C-17, Cat-Nr. 9520, raised against a peptide mapping at the C-terminus of human NE), mouse NE (Santa Cruz Biotechnology Inc., Santa Cruz, CA, USA, polyclonal from goat, M-18, Cat-Nr. 9521, raised against a peptide mapping at the C-terminus of murine NE), BIP (NEB Cell Signaling, Danvers, MA, USA), PDI (NEB Cell Signaling) or GAPDH (Merck Millipore, Darmstadt, Germany). HRP-conjugated seconary antibodies were directed against goat (Dianova, Hamburg, Germany), rabbit (Dianova) or mouse (Sigma-Aldrich, Steinheim, Germany). Detection was performed by chemoluminescence using ECL Prime Western Blotting Detection Reagent (GE Health Care, Solingen, Deutschland) or SuperSignal West Femto Chemiluminescent Substrate (Thermo Scientific, Rochester, NY, USA).

### Flow cytometry analysis of surface markers

Expression of cell surface markers was measured after blocking of unspecific binding sites with CD16/CD32Fc-Block (BD Biosciences, San Jose, CA, USA) by staining cells with anti-Gr-1-APC (BD Biosciences, San Jose, CA, USA, Clone RB6-8C5), anti-Ly6G (Gr-1)-PE (eBioscience, Clone RB6-8C5) anti-CD11b-PE (AbD serotec, Bio-Rad, München, Germany, Clone M1/70), anti-c-kit-APC (eBioscience, San Diego, CA, USA, Clone ACK2), anti-CXCR2-APC (Biolegend, San Diego, CA, USA, Clone TG11/CXCR2) or anti-CXCR4-APC (BD Biosciences, Heidelberg, Germany, Clone 2B11/CXCR4) followed by flow cytometry analysis on a FACS Calibur or Fortessa (BD Biosciences).

### ELISA

For the measurement of tumor necrosis factor (TNF) and interleukin-6 (IL-6) production, supernatants from cell cultures stimulated as indicated were harvested and cytokines were quantified using mouse TNFα and IL-6 ELISA kits (eBioscience), according to the manufacturer’s instructions.

### Adoptive transfer of Hoxb8 progenitor cells

NE^-/-^ Hoxb8 progenitors on C57BL/6 background reconstituted with either wt hNE or the hNE SCN mutant hNEG185R, or expressing empty vector control, were transplanted into syngeneic C57BL/6N wt mice (commercially obtained from Janvier, France), which had been lethally irradiated (2 x 5 Gy) the day before, together with syngeneic bone marrow at a ratio of 10: 1 (5 x 10^6^ Hoxb8 progenitors together with 0,5 x 10^6^ murine bone marrow cells). Mice were sacrificed by cervical dislocation at day 6 after transfer. Blood, bone marrow and spleen were collected and analysed as indicated. All animal experiments performed were approved by the Regierungspräsidium Freiburg (permit number Az 35–9185.81/G-13/009).

## Supporting Information

S1 Fig Analysis of cell morphology of Hoxb8 cells by Giemsa staining. Cytospins of Hoxb8 neutrophil progenitors (NP) and day 4 differentiated neutrophils (day 4 diff.) on 129/Sv background transduced with either empty vector control (pMIGR1), murine NE (mNE), or mNE elastase mutants G185R (mNEG185R) and S97L (mNES97L) were methanol fixed and Giemsa stained. Samples were analysed by brightfield microscopy at a magnification of 63x. Scale bar: 20 μm.
(TIF)

S2 Fig Cell death analysis after ER stress induction by tunicamycin treatment. Day 1 differentiated NE^-/-^ Hoxb8 neutrophils transduced with either empty vector control (pMIGR1), mNE, or mNE mutants G185R (mNE185R) or S97L (mNES97L) were analysed at 0h (A) or treated with tunicamycin (TM; 0,2 μg/ml) for 24h (C), or left untreated (B). Cell death was measured by PI staining and flow cytometry. Data represent mean/SD of 4 independent experiments.
(TIF)

S3 Fig Surface expression of myeloid differentiation markers in progenitors and differentiated neutrophils. Murine Ne^-/-^ progenitors (NP) and day 4 differentiated neutrophils (genetic background C57BL/6) transduced with empty vector (pMIGR1), hNE or hNE mutant G185R (hNeG185R) were stained with fluorescence-conjugated antibodies against Gr-1, CD11b, c-kit, CXCR2 or CXCR4 and analysed by flow cytometry. (A) Gr-1-APC/CD11b-PE double stained NP/day 4 diff. neutrophils. (B) Histograms of NP (dotted line) and day 4 differentiated neutrophils (solid line) showing expression of Gr-1, c-kit, CXCR2 and CXCR4. Data are representative of three independent experiments. (C) Analysis of cell morphology of Hoxb8 cells by Giemsa staining. Cytospins of wt or NE^-/-^ Hoxb8 neutrophil progenitors (NP) and day 4 differentiated neutrophils (day 4 diff.) on C57BL/6 background transduced with either empty vector control (pMIGR1), human NE (hNE) or hNE elastase mutant G185R (hNEG185R) were methanol fixed and Giemsa stained. Samples were analysed by brightfield microscopy at a magnification of 63x. Scale bar: 20 μm. Note that two Gr-1 antibodies have been used (originating from the same clone, but with different conjugations) which show different sensitivities.
(TIF)

S4 Fig Surface expression of myeloid differentiation markers on WT progenitors and differentiated neutrophils. Murine wildtype progenitors (NP) and day 4 differentiated neutrophils (day 4. diff) (genetic background C57BL/6) transduced with mNE mutants G185R (mNEG185R) or hNE mutants G185R (hNEG185R) were stained with fluorescence-conjugated antibodies against Gr-1, CD11b, c-kit, CXCR2 and F4/80 and analyzed by flow cytometry. Histograms of NP (dotted line) and day 4 differentiated neutrophils (solid line) showing expression of Gr-1, CD11b, ckit, CXCR2 and F4/80.
(TIF)

S5 Fig Viability and transfection efficiency of transiently expressing HEK 293-FT. Analysis of transiently transfected HEK 293-FT 64h after transfection with either empty vector (pMiGR1), murine neutrophil elastase (mNE), human neutrophil elastase (hNE) or mNE/hNE mutants G185R (mNEG185R/hNEG185R) or S97L (mNES97L/ hNES97L). (A) Percentage of GFP-positive cells was determined by flow cytometry (B) Cell death was determined by propidium iodide (PI) staining for loss of cell membrane integrity. The percentage of dead cells is indicated. Analysis was done on a FACS Calibur II.
(TIF)

S6 Fig Westernblot showing different NE-specific-Antibodies on 293-FT-cells lysates. Cell lysates from 293-FT cells were analysed 64h after transfection with either empty vector (pMiGR1), human neutrophil elastase (hNE) or hNE mutants G185R (hNEG185R) or S97L (hNES97L) by Western blotting. The membrane was probed/reprobed using several neutrophil elastase-specific antibodies: (A) anti-NE antibody from Merck (Kat-Nr. 481001), (B) human-specific anti-NE from Santa-Cruz (C-17, sc9520), (C) anti-NE antibody (specific for human, mouse and rat) from Santa Cruz (N-18, sc9518). Samples corresponding to 20 μg cell lysate were separated by SDS-PAGE, transferred onto nitrocellulose membranes and probed for the antibodies indicated. GAPDH served as loading control.
(TIF)

S7 Fig Whole western blots relating to Fig 1A (TIF)

S8 Fig Whole western blots relating to Fig 1B (TIF)

S9 Fig Whole western blots relating to Fig 2C (TIF)

S10 Fig Whole western blots relating to Fig 5C (TIF)

S11 Fig Relative quantification of NE protein expression in differentiating Hoxb8 cells. Wt Hoxb8 cells (B6 background) were induced to undergo differentiation by estrogen withdrawal and were monitored for NE expression daily from day 0 to day 4. Cells were harvested at the indicated time points, washed once with PBS and immediately lysed and boiled in Laemmli buffer. Whole-cell lysates were separated by SDS-PAGE, transferred onto nitrocellulose membrane and probed for mNE. GAPDH served as loading control. NE protein expression levels for each experiment were quantified using LabImage 1D analysis software (Intas). Data were normalized to GADPH expression and calculated relative to day 0. Shown are three independent experiments.
(TIF)
