# Supplementary figures and images for: Characterisation of Neutropenia-Associated Neutrophil Elastase Mutations in a Murine Differentiation Model In Vitro and In Vivo
Source: PLoS One. 2016 Dec 12;11(12):e0168055. doi: 10.1371/journal.pone.0168055 (PMC5152902; doi:10.1371/journal.pone.0168055)

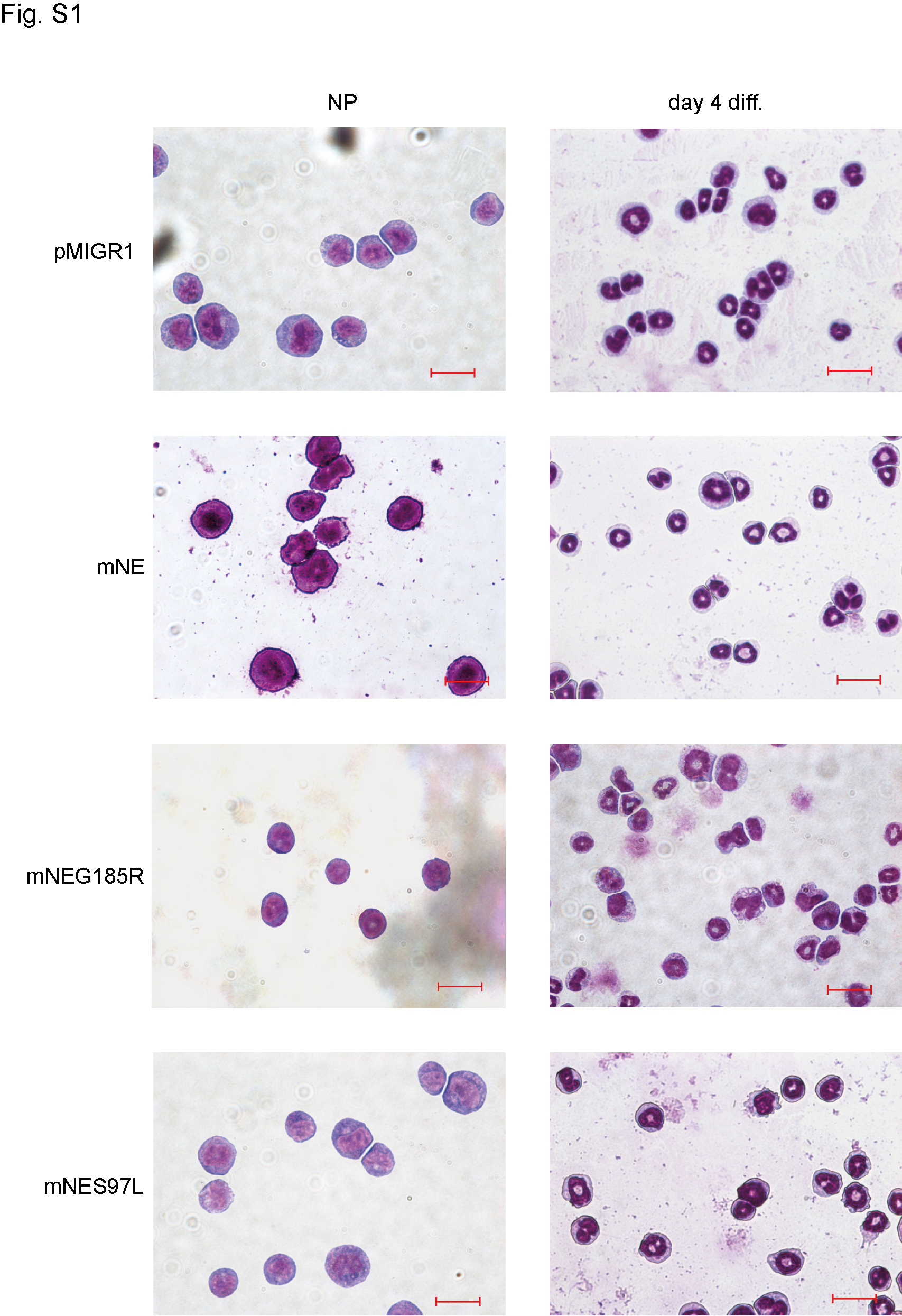

Supplement: S1 Fig — Cytospins of Hoxb8 neutrophil progenitors (NP) and day 4 differentiated neutrophils (day 4 diff.) on 129/Sv background transduced with either empty vector control (pMIGR1), murine NE (mNE), or mNE elastase mutants G185R (mNEG185R) and S97L (mNES97L) were methanol fixed and Giemsa stained. Samples were analysed by brightfield microscopy at a magnification of 63x. Scale bar: 20 μm. (TIF) [file pone.0168055.s001.tif]

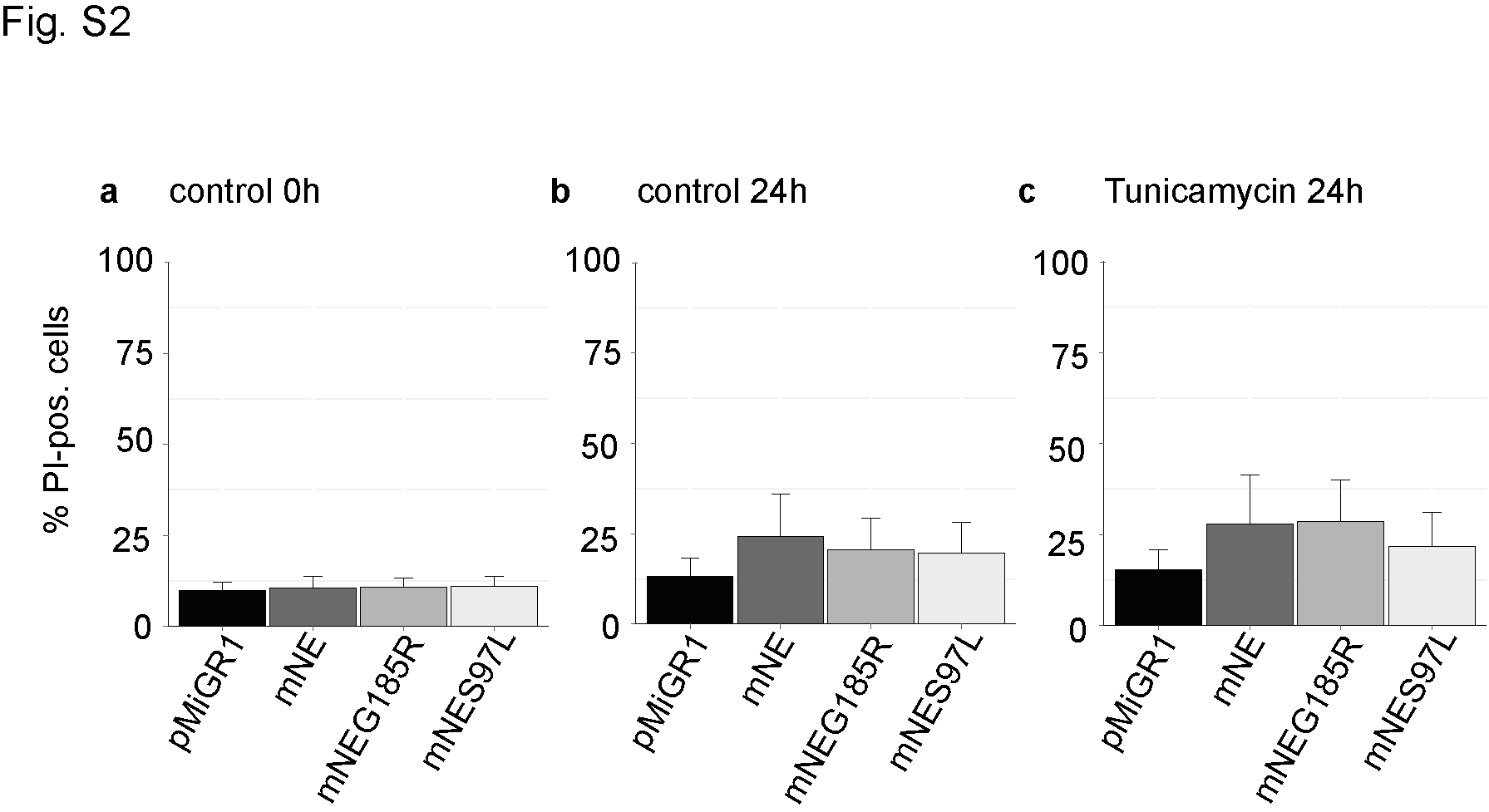

Supplement: S2 Fig — Day 1 differentiated NE-/- Hoxb8 neutrophils transduced with either empty vector control (pMIGR1), mNE, or mNE mutants G185R (mNE185R) or S97L (mNES97L) were analysed at 0h (A) or treated with tunicamycin (TM; 0,2 μg/ml) for 24h (C), or left untreated (B). Cell death was measured by PI staining and flow cytometry. Data represent mean/SD of 4 independent experiments. (TIF) [file pone.0168055.s002.tif]

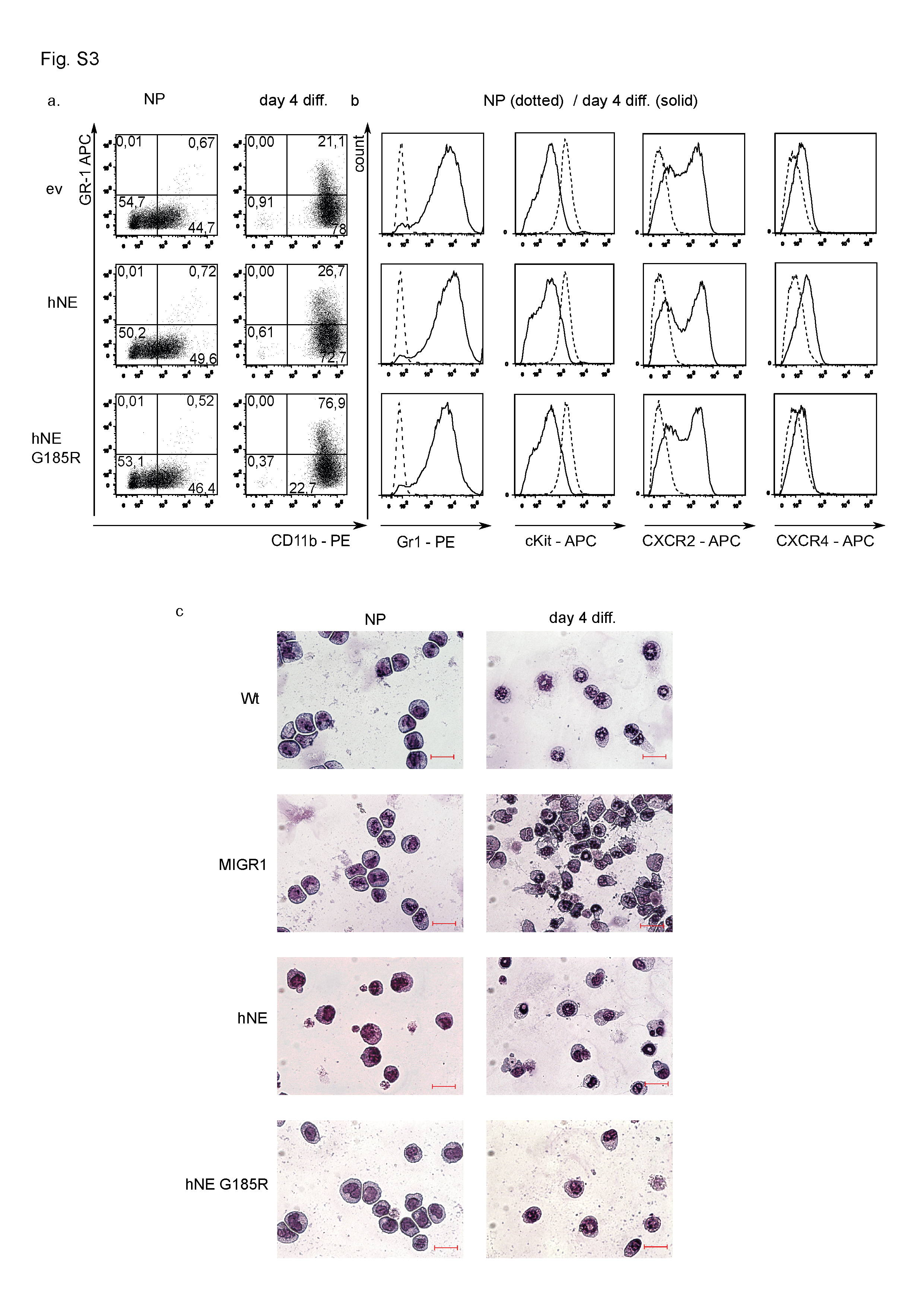

Supplement: S3 Fig — Murine Ne-/- progenitors (NP) and day 4 differentiated neutrophils (genetic background C57BL/6) transduced with empty vector (pMIGR1), hNE or hNE mutant G185R (hNeG185R) were stained with fluorescence-conjugated antibodies against Gr-1, CD11b, c-kit, CXCR2 or CXCR4 and analysed by flow cytometry. (A) Gr-1-APC/CD11b-PE double stained NP/day 4 diff. neutrophils. (B) Histograms of NP (dotted line) and day 4 differentiated neutrophils (solid line) showing expression of Gr-1, c-kit, CXCR2 and CXCR4. Data are representative of three independent experiments. (C) Analysis of cell morphology of Hoxb8 cells by Giemsa staining. Cytospins of wt or NE-/- Hoxb8 neutrophil progenitors (NP) and day 4 differentiated neutrophils (day 4 diff.) on C57BL/6 background transduced with either empty vector control (pMIGR1), human NE (hNE) or hNE elastase mutant G185R (hNEG185R) were methanol fixed and Giemsa stained. Samples were analysed by brightfield microscopy at a magnification of 63x. Scale bar: 20 μm. Note that two Gr-1 antibodies have been used (originating from the same clone, but with different conjugations) which show different sensitivities. (TIF) [file pone.0168055.s003.tif]

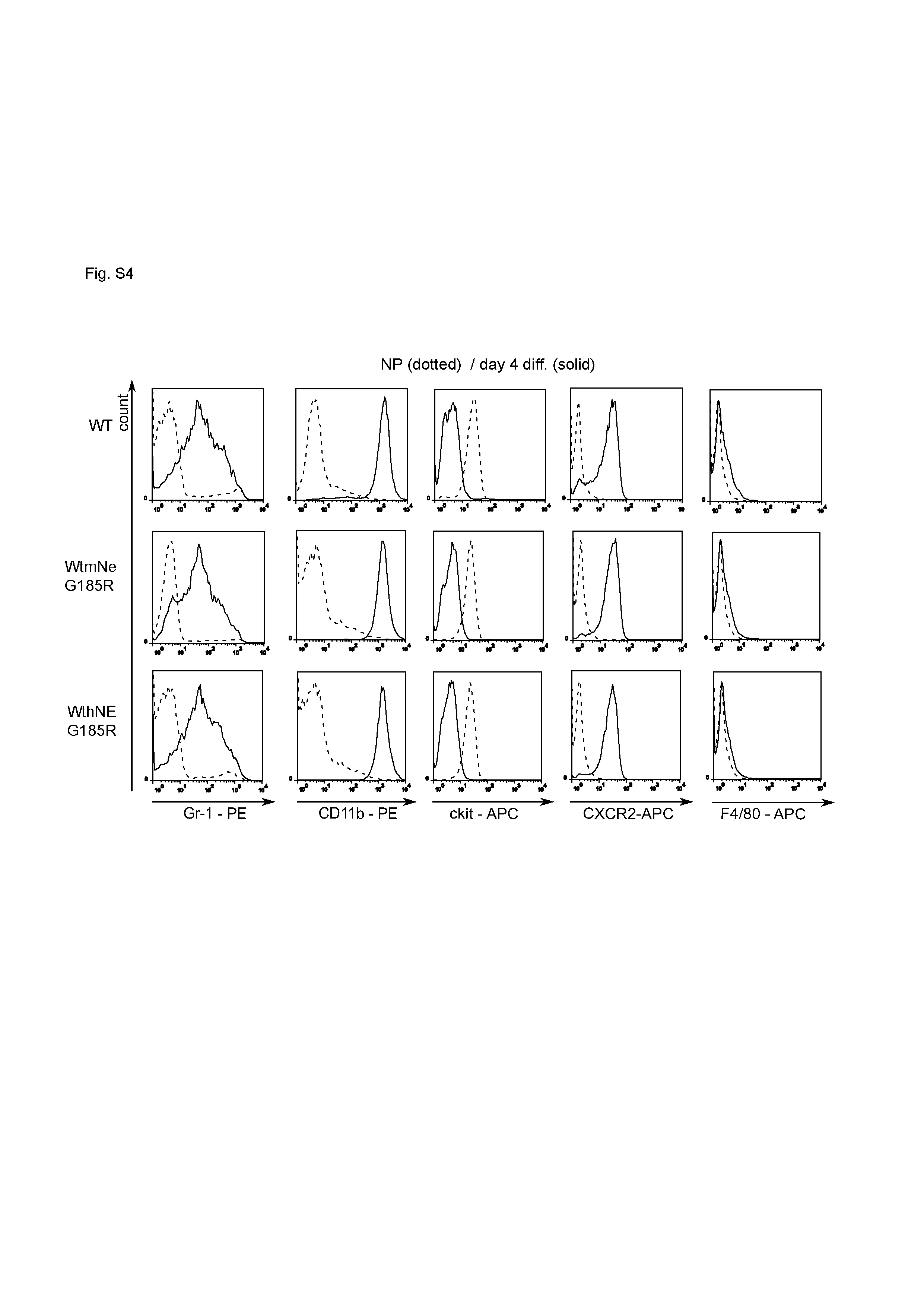

Supplement: S4 Fig — Murine wildtype progenitors (NP) and day 4 differentiated neutrophils (day 4. diff) (genetic background C57BL/6) transduced with mNE mutants G185R (mNEG185R) or hNE mutants G185R (hNEG185R) were stained with fluorescence-conjugated antibodies against Gr-1, CD11b, c-kit, CXCR2 and F4/80 and analyzed by flow cytometry. Histograms of NP (dotted line) and day 4 differentiated neutrophils (solid line) showing expression of Gr-1, CD11b, ckit, CXCR2 and F4/80. (TIF) [file pone.0168055.s004.tif]

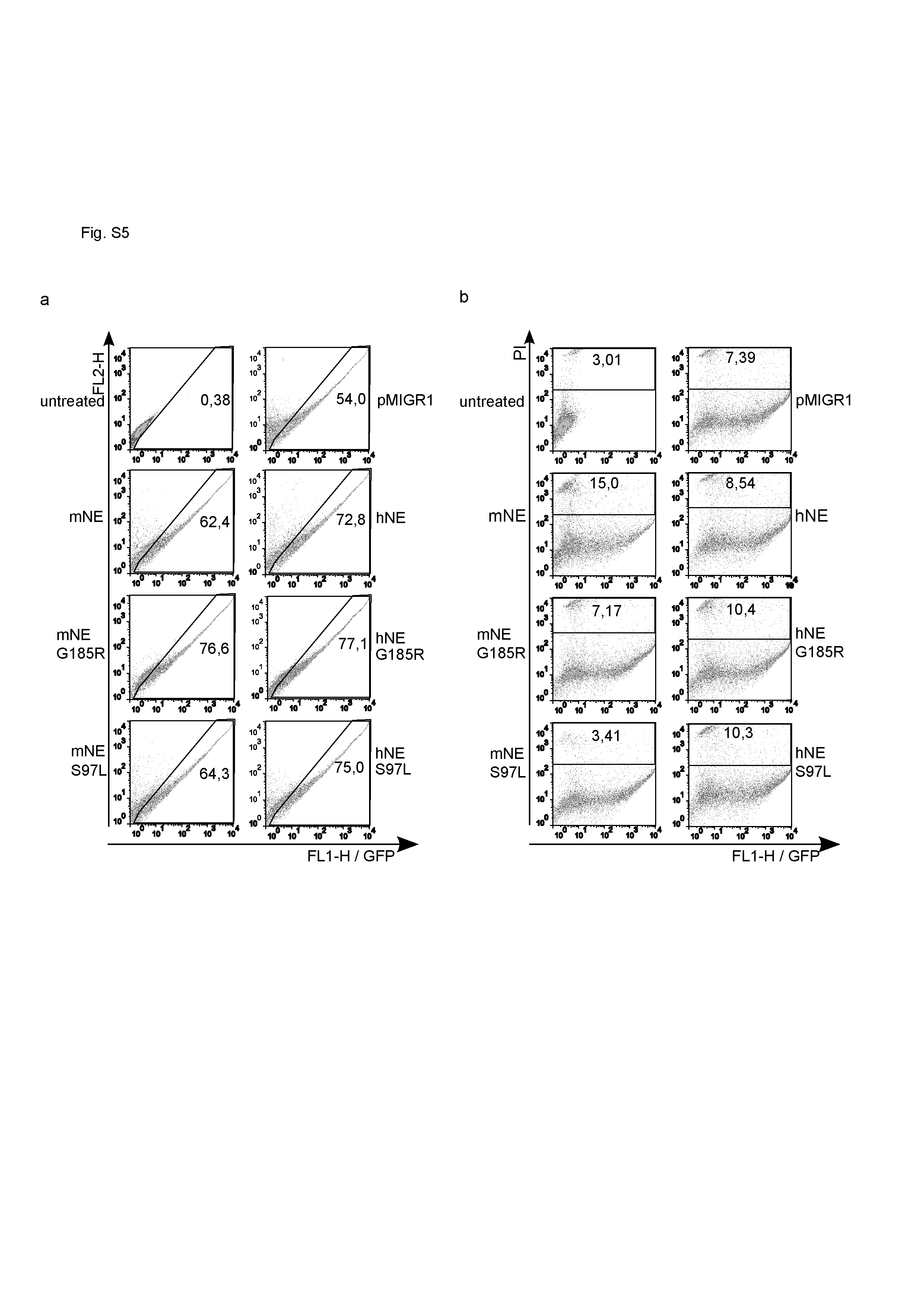

Supplement: S5 Fig — Analysis of transiently transfected HEK 293-FT 64h after transfection with either empty vector (pMiGR1), murine neutrophil elastase (mNE), human neutrophil elastase (hNE) or mNE/hNE mutants G185R (mNEG185R/hNEG185R) or S97L (mNES97L/ hNES97L). (A) Percentage of GFP-positive cells was determined by flow cytometry (B) Cell death was determined by propidium iodide (PI) staining for loss of cell membrane integrity. The percentage of dead cells is indicated. Analysis was done on a FACS Calibur II. (TIF) [file pone.0168055.s005.tif]

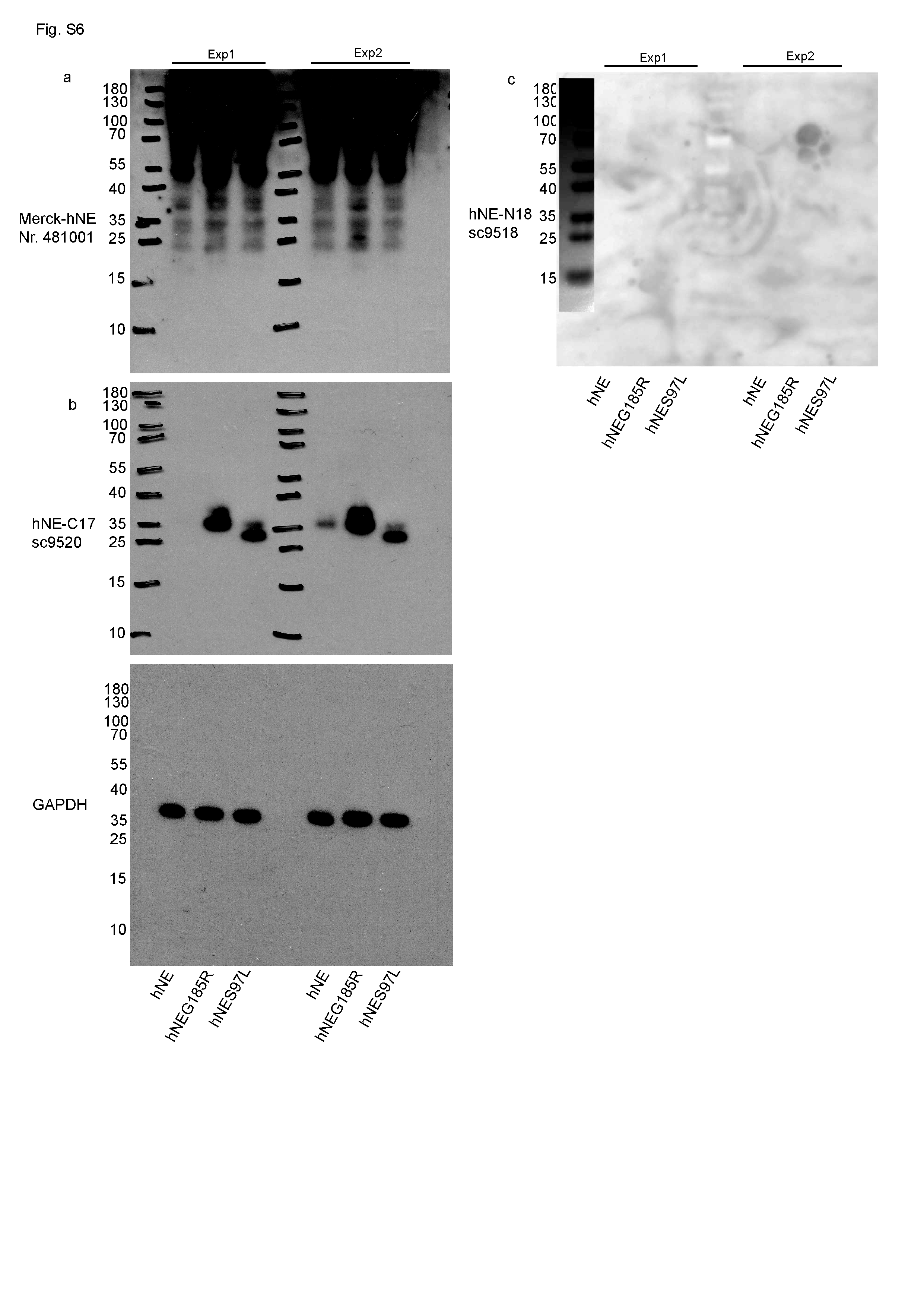

Supplement: S6 Fig — Cell lysates from 293-FT cells were analysed 64h after transfection with either empty vector (pMiGR1), human neutrophil elastase (hNE) or hNE mutants G185R (hNEG185R) or S97L (hNES97L) by Western blotting. The membrane was probed/reprobed using several neutrophil elastase-specific antibodies: (A) anti-NE antibody from Merck (Kat-Nr. 481001), (B) human-specific anti-NE from Santa-Cruz (C-17, sc9520), (C) anti-NE antibody (specific for human, mouse and rat) from Santa Cruz (N-18, sc9518). Samples corresponding to 20 μg cell lysate were separated by SDS-PAGE, transferred onto nitrocellulose membranes and probed for the antibodies indicated. GAPDH served as loading control. (TIF) [file pone.0168055.s006.tif]

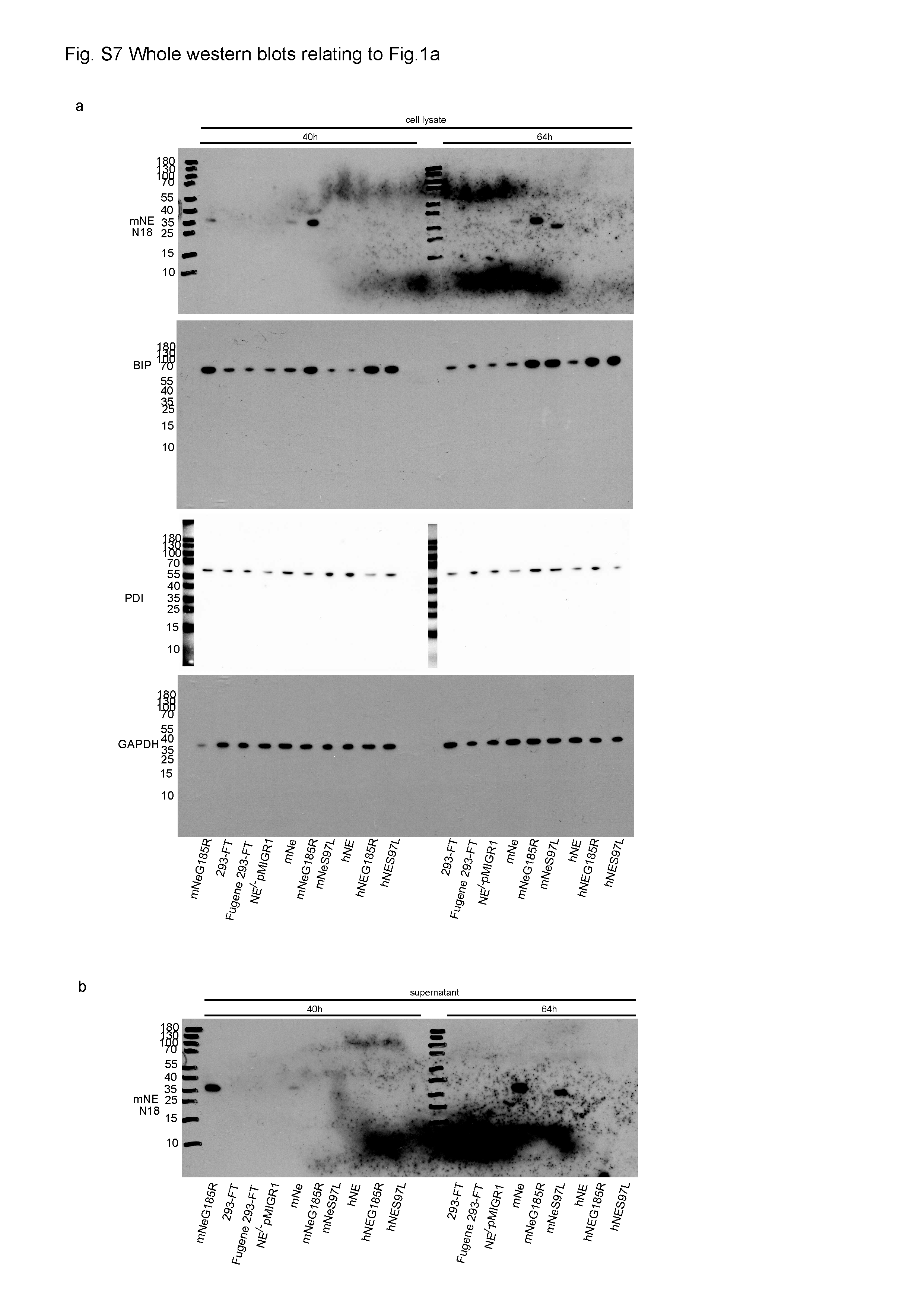

Supplement: S7 Fig — (TIF) [file pone.0168055.s007.tif]

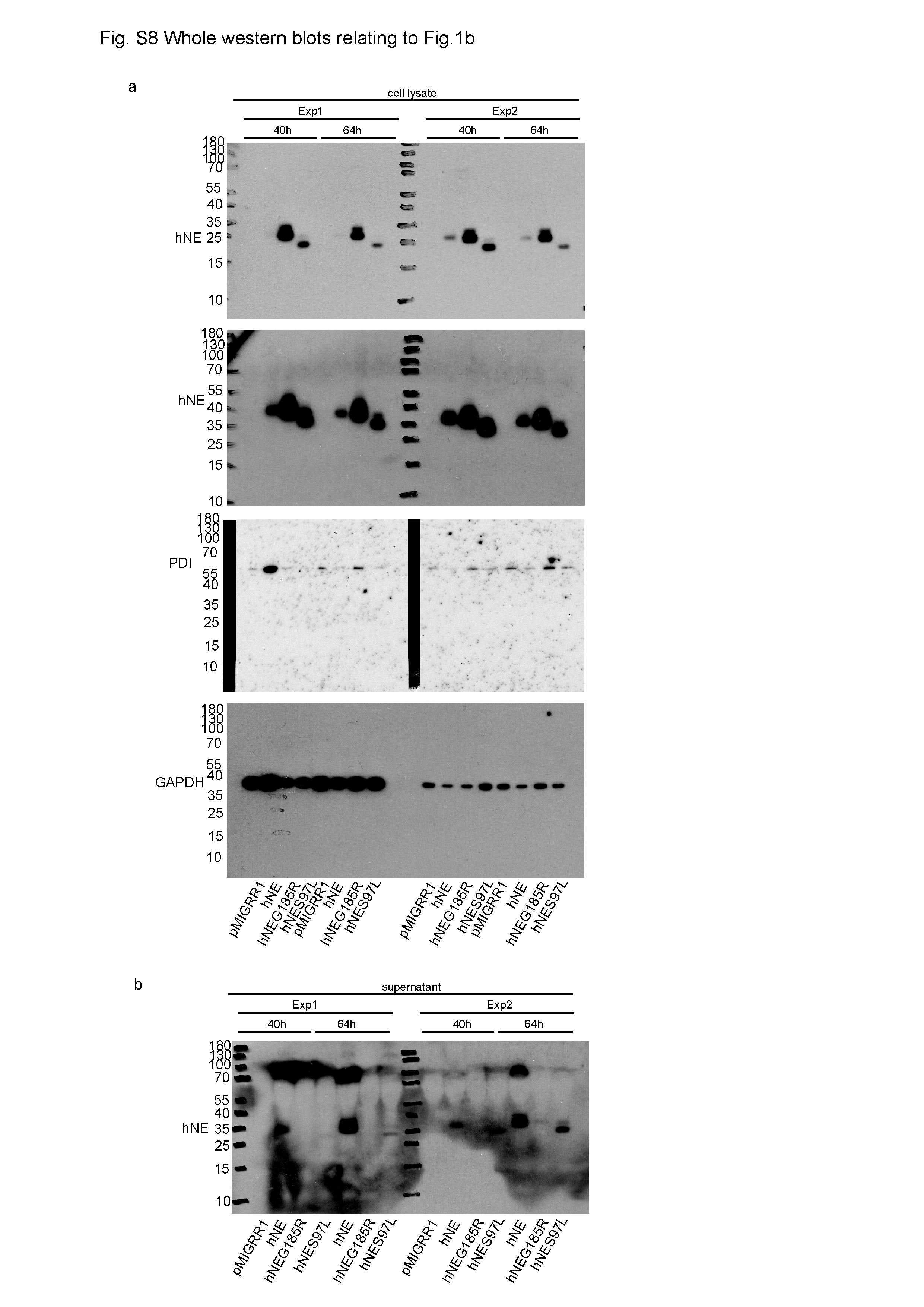

Supplement: S8 Fig — (TIF) [file pone.0168055.s008.tif]

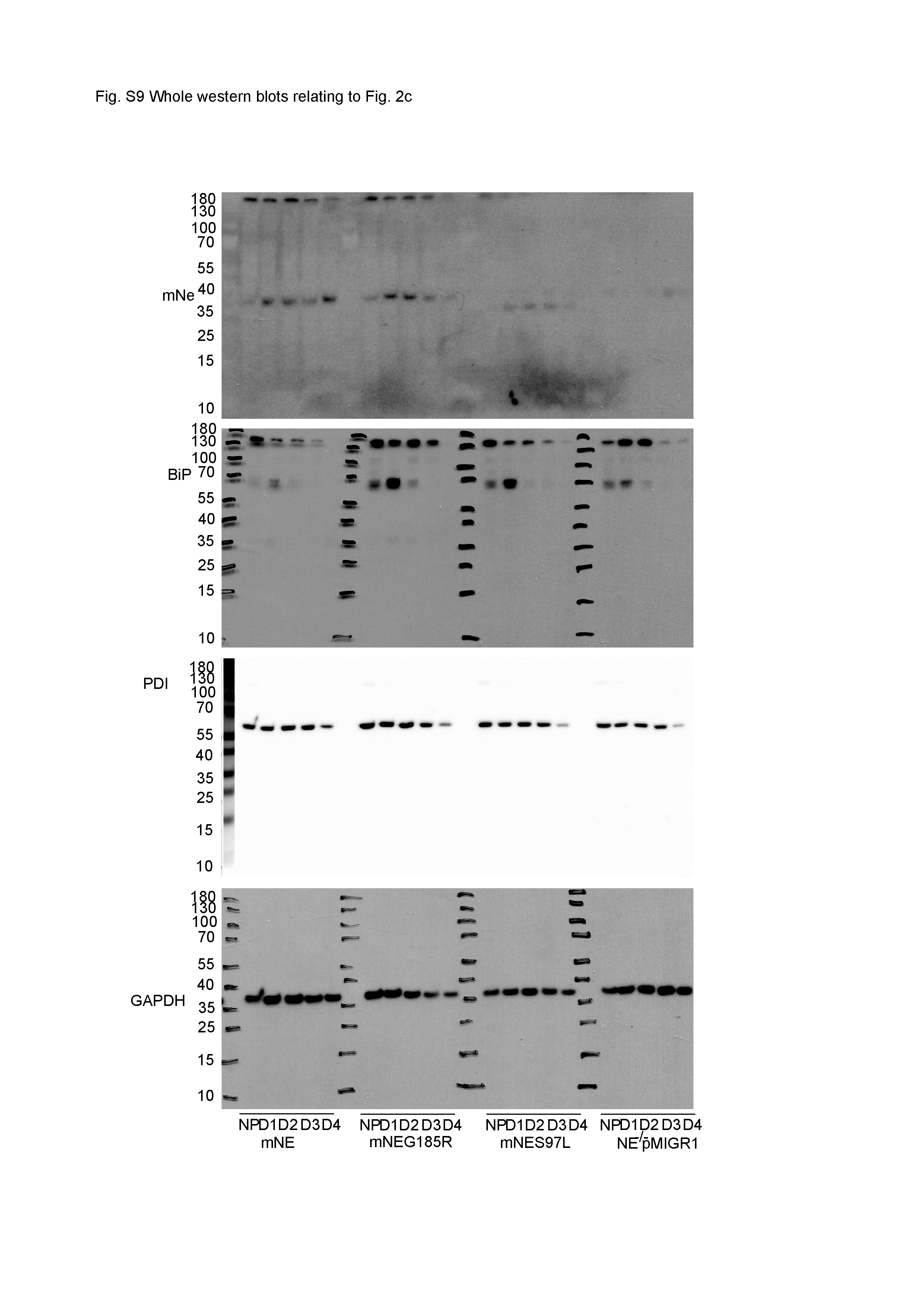

Supplement: S9 Fig — (TIF) [file pone.0168055.s009.tif]

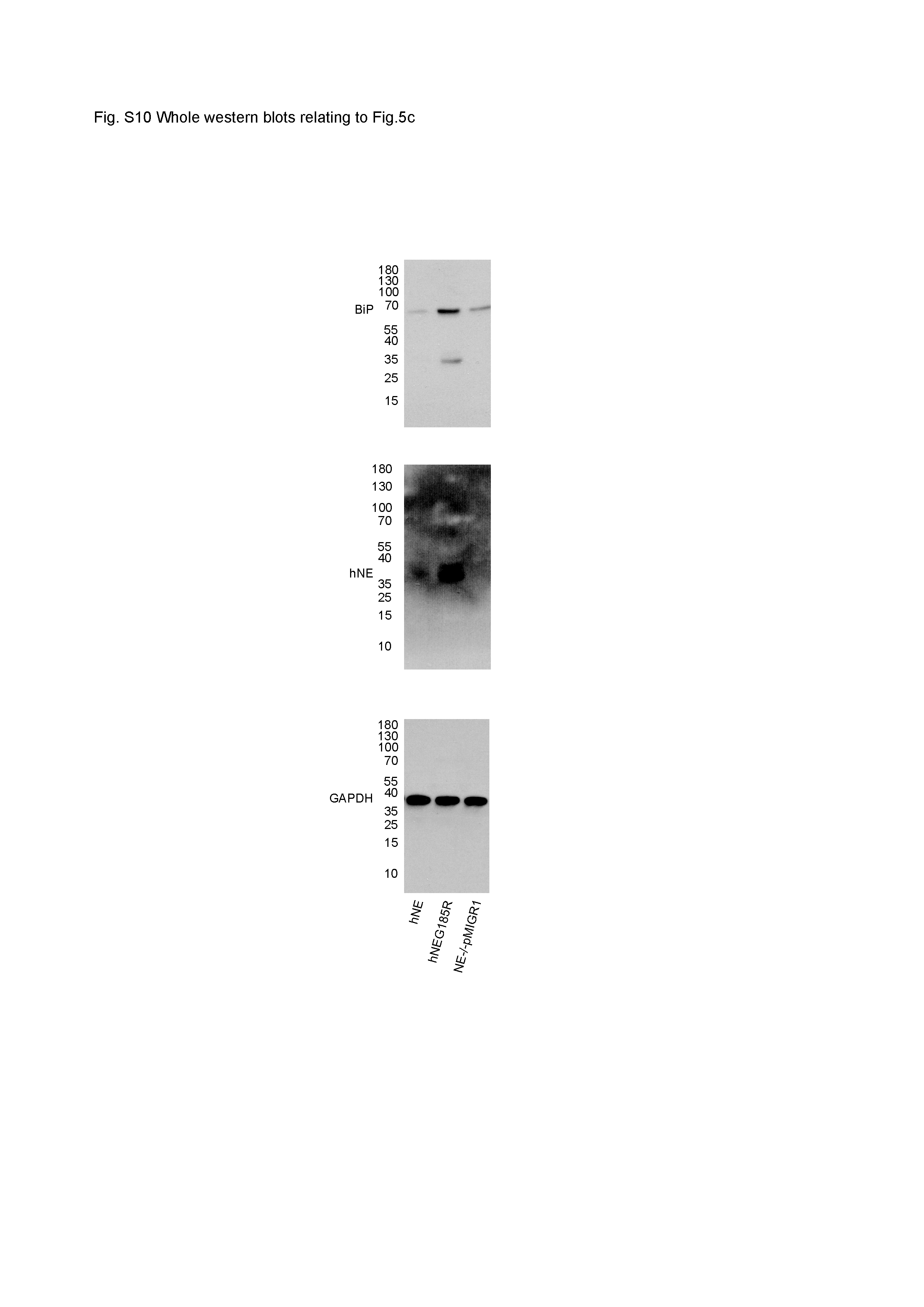

Supplement: S10 Fig — (TIF) [file pone.0168055.s010.tif]

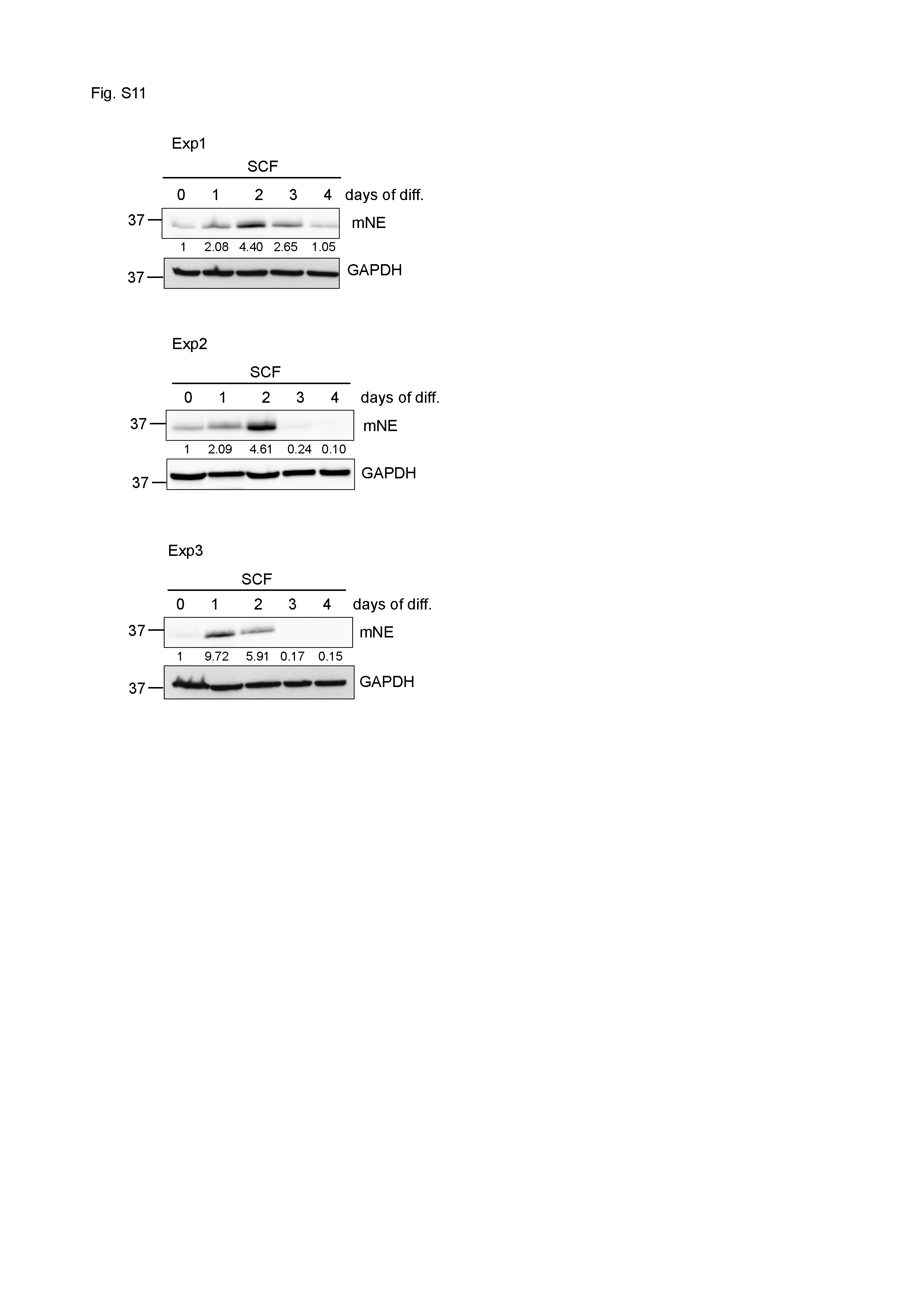

Supplement: S11 Fig — Wt Hoxb8 cells (B6 background) were induced to undergo differentiation by estrogen withdrawal and were monitored for NE expression daily from day 0 to day 4. Cells were harvested at the indicated time points, washed once with PBS and immediately lysed and boiled in Laemmli buffer. Whole-cell lysates were separated by SDS-PAGE, transferred onto nitrocellulose membrane and probed for mNE. GAPDH served as loading control. NE protein expression levels for each experiment were quantified using LabImage 1D analysis software (Intas). Data were normalized to GADPH expression and calculated relative to day 0. Shown are three independent experiments. (TIF) [file pone.0168055.s011.tif]
